# Responding to the COVID-19 pandemic through global partnerships: the case of the HRP Alliance for research capacity strengthening

**DOI:** 10.1186/s12978-026-02342-9

**Published:** 2026-07-23

**Authors:** Vanessa Brizuela, Mehr G. Shah, Elizabeth Fine, Luis Bahamondes, Evelyn Gitau, Seni Kouanda, Pisake Lumbiganon, Thi Thuy Hanh Nguyen, Sarah Saleem, Kwasi Torpey, Anna Thorson, the WHO HRP Social Science Research Team, Jose Guilherme Cecatti, Vilma Zotareli, Rachel E Soeiro, Karayna G Fernandes, Mariana B Rogerio, Samira M Haddad, Silvana F Bento, Karla S Padua, Aline Munezero, Charles M Charles, Montas Laporte, Eunice Chomi, Flore Marie Gisèle Donessouné, Armel Sogo, Kun Tang, Yueping Guo, Hanxiyue Zhang, Yifan Zhu, Ge Yang, Chunxiao Peng, Xizhuo Xie, Hao Wang, Deda Ogum Alangea, Emefa Judith Modey, Adom Manu, Ernest T. Maya, Rozina Karmaliani, Laila Ladak, Arusa Lakhani, Marina Baig, Yasmin Parpio, Salima Somani, Jen Sothornwit, Nampet Jampathong, Somporn Rungreangkulkij, Marleen Temmerman, Ferdinand Okwaro, Abdu Mohiddin, Massimo Mirandola, Maddalena Cordioli, Alessia Savoldi, Simone Garzon, Stefano Uccella, Ranieri Poli, Nigel Sherriff, Alexandra Sawyer, Catherine Aicken, Jörg W Huber, Jaime Vera, Deborah Williams, Moazzam Ali, Caron Kim, Hamsadvani Kuganantham, Grace Kapustianyk, Igor Toskin, Soe Soe Thwin, Joy Jerop Chebet, Hugo Gamerro Abrego, Armando Seuc, Gabriela García Camacho

**Affiliations:** 1 UNDP/UNFPA/UNICEF/WHO/World Bank Special Programme of Research, Development and Research Training in Human Reproduction (HRP), Department of Sexual, Reproductive, Maternal, Child, Adolescent Health and Ageing, World Health Organization, Avenue Appia 20, Geneva, 1211 Switzerland; 2 Department of Obstetrics and Gynaecology, Faculty of Medical Sciences, University of Campinas, Campinas, SP Brazil; 3 African Population and Health Research Center, Nairobi, Kenya; 4 Institut de Recherche en Sciences de la Santé (IRSS), Ouagadougou, Burkina Faso; 5 Department of Obstetrics and Gynaecology, Faculty of Medicine, Khon Kaen University, Khon Kaen, Thailand; 6 Institute for Preventive Medicine and Public Health, Hanoi Medical University, Hanoi, Vietnam; 7 Department of Community Health Sciences, Aga Khan University, Karachi, Pakistan; 8 Department of Population, Family and Reproductive Health, School of Public Health, University of Ghana, Accra, Ghana

**Keywords:** Sexual and reproductive health, Research, Research capacity strengthening, COVID-19, Emergency preparedness and response

## Abstract

**Background:**

The UNDP/UNFPA/UNICEF/WHO/World Bank Special Programme of Research, Development and Research Training in Human Reproduction (HRP) has a mandate to lead in sexual and reproductive health and rights research and to support research capacity strengthening. Starting in 2016, it did so through supporting the latter through a large network of research institutions called the HRP Alliance. This commentary highlights the work of the HRP Alliance in response to the coronavirus disease (COVID-19) pandemic.

**Main body:**

The onset of the COVID-19 pandemic prompted the HRP Alliance to adapt the way they had been working. HRP Alliance research capacity strengthening hubs actively contributed to developing a research agenda based on WHO’s research and development blueprint and country-specific needs. They also took on leading roles in developing, adapting to each country/setting, and implementing research projects aimed at understanding how the COVID-19 pandemic was affecting SRHR in different contexts and income settings. These studies provided opportunities for early career researchers and students to lead in project management, study implementation, training, and data analysis. Through a network of nimble research institutions, the HRP Alliance collaborated to generate evidence on the impact of COVID-19 on pregnancy, pregnancy outcomes, access to SRHR services, gender-based violence, and abortion care. The success of implementing these research response mechanisms and developing global networks of research and healthcare institutions, provided solid ground upon which to build SRHR research responses to future pandemics and other emerging diseases.

**Conclusion:**

Adequate readiness and response to global health emergencies require high quality and timely evidence generation. Global networks of research partner institutions, brought together through research capacity strengthening initiatives, can provide a fruitful platform ready and able to swiftly respond. However, inherent power imbalances and challenges to equitable partnerships need to be considered to ensure sustainable ways of working together.

## Background

**Fig. 1:**
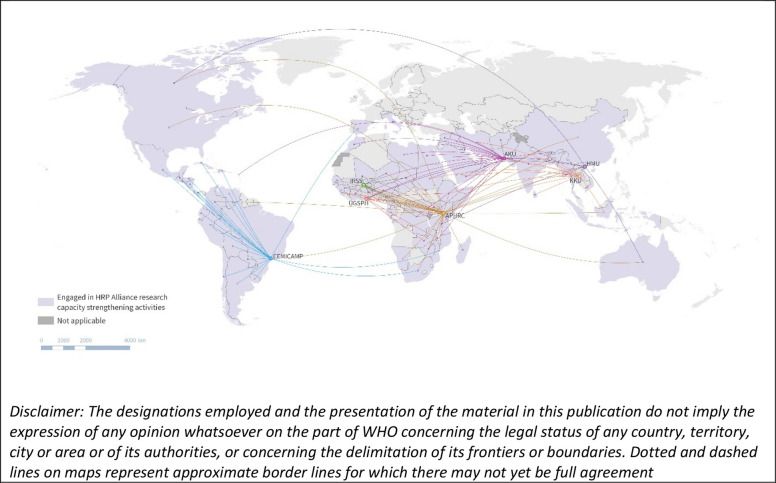
World map depicting the HRP Alliance hubs (indicated with coloured dots) and their spokes across the regions

Strong research teams are necessary to respond to health priority needs and evidence generation. Since its creation in 1972, the UNDP/UNFPA/UNICEF/WHO/World Bank Special Programme of Research, Development and Research Training in Human Reproduction (HRP) is unique in its dual mandate to lead in sexual and reproductive health and rights (SRHR) research and to support research capacity strengthening (RCS). Starting in 2016, HRP supported institutional capacity strengthening through the HRP Alliance for RCS [1]. The HRP Alliance was a global network for SRHR research, which included WHO country and regional offices, WHO Collaborating Centres, HRP Alliance grantees, institutions that collaborated with HRP in global research projects (HRP Alliance spokes), and central to this network, the HRP Alliance RCS regional hubs. These hubs were selected through competitive, open calls, and were mandated with leading SRHR RCS efforts in their regions or sub-regions, responding to local SRHR and HRP-led research needs and priorities and in line with their institutional strengths (Fig. 1). To this end, between 2017 and 2025, the HRP Alliance supported over 5,000 participants to attend online or in-person short courses and workshops on SRHR topics and research methodology, and 54 doctoral and 124 master’s students in completing their research degrees in SRHR-related topics, hosted a global mentorship programme for over 50 early career women SRHR researchers, and supported dozens of researchers in completing analyses and publications using data from HRP research studies [2, 3]. Further to this, the HRP Alliance hubs strengthened their regional networks by bringing spokes together for joint research prioritization, grant proposal writing, and collaboration, among others. Additionally, the seven HRP Alliance regional hubs brought collaborators together to develop research partnerships and consortia which were leveraged to support research responding to emerging health needs, such as the Zika virus epidemic and migration crises [4–7]. In 2016, the HRP Alliance partnered in joint response to the Zika pandemic and from 2020 to 2024 they became integral partners in HRP’s response to the COVID-19 pandemic. This commentary, together with the articles included in this supplement, highlights and celebrates the capacity of the network to rapidly respond to the urgent need to generate evidence relating to how COVID-19 affected different aspects of SRHR, while continuing its work in RCS.

## Main text

### Emergency response

The onset of the COVID-19 pandemic prompted the HRP Alliance to quickly adapt the way they had been working until then and to use the network to swiftly come together to conduct research responding to the pandemic’s emerging needs. Early in 2020, as COVID-19 was first declared a public health emergency of international concern [8], HRP brought together staff and partners to develop a cohesive response with regards to sexual and reproductive health research [9]. This included the HRP Alliance hubs that actively contributed to developing the research agenda. Using experiences from prior epidemics and health emergencies, the group developed generic research protocols to support research teams globally to collect relevant and critical data on the effects of COVID-19 on sexual and reproductive health outcomes [10, 11]. The work of this group complemented and contributed to the broader WHO emergency response through the research and development blueprint on COVID-19 and the global clinical platform [12–14]. Specifically, the HRP Alliance, through the leadership of the hubs and the support from the HRP Alliance Secretariat at WHO headquarters, took leading roles in developing, adapting to each country/setting, and implementing research projects aimed at understanding how the COVID-19 pandemic was affecting SRHR in different contexts and income settings. While this approach did not change the essence of the HRP Alliance and its anchor in research capacity strengthening, it fostered a different kind of collaboration and leveraged on the hubs’ regional leadership to rapidly activate a network of researchers. The HRP Alliance, along with its hubs, research partners, and WHO Collaborating Centres, quickly became a key partner in facilitating and contributing to the following:

- developing the generic protocol and bringing together cross-national collaborators in implementing the WHO/HRP Unity [15] prospective cohort study investigating maternal, pregnancy and neonatal outcomes for women and neonates infected with SARS-CoV-2 [16];
- developing two generic protocols for publication and further country adaptation and implementation on (i) a health systems analysis and evaluation of barriers to availability, utilization, and readiness of sexual and reproductive health services in COVID-19-affected areas using mixed-methods and (ii) issues related to pregnancy, pregnancy prevention, and abortion in the context of the COVID-19 pandemic using qualitative methods [10, 11];
- synthesizing the evidence, as it became available, through living systematic reviews on clinical aspects, treatment options, global variations, and mother-to-child transmission of SARS-CoV-2 among pregnant and recently pregnant women and their newborns [17–20];
- developing the protocol and implementing a multi-country study exploring knowledge, attitudes, and practices related to COVID-19 vaccine decision-making among pregnant and recently pregnant women [21].

Further to these examples, HRP Alliance partners coordinated regional and national networks to conduct studies relating to the COVID-19 pandemic, bringing together clinicians, academics, and policymakers [22–25]. The HRP Alliance hubs played central roles in national research response, e.g.: being part of the country pandemic response task force in Burkina Faso, providing the evidence base for revised policies on vaccination among pregnant women in Ghana, and creating a network to collect data among pregnant women and COVID-19 in Brazil. The work completed with the support and leadership of the HRP Alliance hubs and research partners provided opportunities for early career researchers and students to lead in project management, study implementation, training, and data analysis further advancing the goal and mandate to embed RCS activities [26–33].

Additional efforts of this collaboration are evident in the papers included in this supplement: four HRP Alliance hubs were engaged in the development of the protocol and implementation of the mixed-methods, multi-country studies that were implemented in their countries and included in this series [34–41]. This supplement includes findings from studies focused on disruptions to sexual and reproductive health services including access and provision of contraception, and abortion services during the pandemic as well as the psychosocial impact of the pandemic on healthcare workers, including health system response to address these issues. All four hubs implementing this protocol [10] offered opportunities for leadership to junior faculty as well as first-hand learning experiences to students completing master’s and/or doctoral degrees in their institutions. The inclusion of partners beyond the HRP Alliance hubs provide additional support to the idea that a strong network of research partners brought together to respond to emerging health needs can prove beneficial to generate the evidence and strengthen the capacity to do so.

### Adapted research capacity strengthening during COVID-19

The COVID-19 pandemic-imposed restrictions to people’s mobility and interactions in efforts to diminish infection transmission required novel -and adaptable- ways in which to conduct research and RCS activities. For example, trainings and degree programmes offered through the HRP Alliance shifted online and remote data collection methods through Voice over Internet Protocols (VoIP) technologies (e.g., Zoom, Microsoft Teams) were utilized. Support to institutions within the network was done solely over the phone or using online technologies.

Among the changes that had to be incorporated when conducting research were adherence to infection prevention and control protocols for studies requiring in-person data collection or the use of online platforms for others, including qualitative interviewing. Local ethics review committees had to quickly change their priorities to both adapt to the emerging health needs as well as to accelerate processes. In some cases, however, they were unable to fully and quickly respond. These changes had to be considered for studies beyond those included in this supplement and those focusing specifically on COVID-19. An example being the HRP Alliance supported project responding to the migration crisis in the Americas which had to swiftly adapt data collection methods and research questions [7].

In addition to research, training programmes and some master’s degree programmes moved to be completely online and students were able to complete their studies from their home countries, abiding by travel restrictions. This allowed for student geographical diversity that would have otherwise been restricted due to immigration bottlenecks. However, these shifts were not met without challenges. While some institutions successfully moved their degree programmes online, others encountered resistance to adapt programmes despite having had successful examples, which reduced opportunities for international scholars to pursue research education as part of the broader HRP Alliance network.

While many of these changes were temporary, demonstrating an agility to shift as needed, some of these were adopted as routine. Many training courses moved permanently online, even for topics that are sensitive or require group work including training on qualitative and quantitative research methods and values clarification and attitudes transformation training. The use of online technology for data collection is now being used more consistently given its flexibility, cost-effectiveness, and the improvement of technologies and platforms that ensure confidentiality. While the shifts to online learning were based largely on necessity, they proved to be beneficial both in expanding access to individuals who may not have previously been able to access these resources as well as in effectiveness [42]. With the end of the emergent period of the COVID-19 pandemic, it remains important to assess which changes would be good to maintain.

### Lessons learned

The lessons learned and the adaptations that were implemented because of the COVID-19 pandemic provided examples of how to prepare and respond to future health emergencies while fostering opportunities for increased research capacity. The HRP Alliance, through its network of research institutions (many with experience working together), effectively collaborated to generate crucial evidence elucidating the impact of a rapidly emerging and growing infectious agent affecting pregnancy, pregnancy outcomes, access to SRHR services, gender-based violence, and abortion care. The success of implementing these research response mechanisms and developing global networks of research and healthcare institutions, provides solid ground upon which to build SRHR research responses to future pandemics and other emerging diseases. Furthermore, generating the standardized protocols have equipped them with the tools for a swifter research response to the next pandemic.

Notwithstanding, critical changes to how research is designed, planned, and implemented are needed. Many ethics and scientific review panels did not have the technical capability, nor did they have the human resource capacity to opportunely deal with these changes and adaptations and were not able to alter lengthy ethical approval processes even under exceptional circumstances. There is a need for increased investment in developing the capacity of ethics and scientific review panels [43–45]. Similarly, traditional research data collection methods were forced to shift and change given precautions needed when faced with a virus of which we knew little. Despite widespread use of online technologies for data collection and training, these are not always available everywhere or their quality is limited, and they pose significant challenges for multi-country research spanning many different time zones.

Importantly, while maintaining and sustaining networks takes time, resources, and concrete actions and goals that bring all actors together, and while oftentimes research networks struggle to survive once funding ends or without the support of a central, coordinating figure (e.g., INCLEN, LatinCLEN, COHRED), the foundational work of networks can still provide useful lessons learned [46–48]. Even as HRP moves towards a different approach to research capacity strengthening, the relationships built over years of working together and the successes of the hubs in fostering the regional networks have the potential to live on beyond the scope of the HRP Alliance. What the pandemic shed light on was that having a readily available network was critical to rapidly responding to the needs. HRP is harnessing this power through continued efforts at protecting and expanding such networks. These partnerships built on trust and collaboration can provide synergies for research and research capacity strengthening.

## Conclusions

Global networks of research partner institutions that are ready and able to swiftly respond to health emergencies are critical to ensure that evidence generation occurs in a timely fashion with the capacity to result in public health policy to uphold sexual and reproductive health and rights. Yet, emergent needs also pose significant challenges to research institutions, some that reflect and expose inherent power asymmetries in research and research capacity. It is therefore important to ensure that networks are built on trust and collaboration.

## Data Availability

Data sharing is not applicable to this article as no datasets were generated or analysed during the current study.

## Abbreviations

COVID-19: Coronavirus disease 2019
HRP: UNDP/UNFPA/UNICEF/WHO/World Bank Special Programme of Research, Development, and Research Training in Human Reproduction
RCS: Research capacity strengthening
SARS-CoV-2: Severe acute respiratory syndrome coronavirus 2
SRHR: Sexual and reproductive health and rights

## References

[1] Adanu R, Bahamondes L, Brizuela V, Gitau E, Kouanda S, Lumbiganon P, et al. Strengthening research capacity through regional partners: the HRP Alliance at the World Health Organization. Reprod Health. 2020;17:131. 10.1186/s12978-020-00965-0. 32847605 10.1186/s12978-020-00965-0 PMC7448306

[2] HRP Alliance: Celebrating five years of research capacity strengthening and learning. 2023. https://www.youtube.com/watch?v=hCg1PGwIt4E. Cited 19 Jul 2023. Accessed 19 Jul 2023.

[3] Brizuela V, Chebet JJ, Thorson A. Supporting early-career women researchers: lessons from a global mentorship programme. Glob Health Action. 2023;16. https://www.tandfonline.com/doi/epdf/10.1080/16549716.2022.2162228?needAccess=true&role=button. Cited 8 Mar 2023. Accessed 8 Mar 2023. 10.1080/16549716.2022.2162228 PMC9888473 36705071

[4] Cecatti JG, Brizuela V. Building opportunities during the Zika epidemic in the Americas: The case for strengthening research capacity. Int J Gynecol Obstet. 2020;148:e1-75. 10.1002/ijgo.13053.

[5] Thorson A, Aslanyan G, Brizuela V, Perez F, León RGPde, Reeder JC, et al. Research and research capacity strengthening in the context of an emerging epidemic: Zika virus in Latin America. Int J Gynaecol Obstet. 2020;148:1–3. 10.1002/ijgo.13040. 31975399 10.1002/ijgo.13040

[6] Brizuela V, Bahamondes L, Gómez Ponce de León R, Aslanyan G, Feletto M, Bonet M, et al. Strengthening locally led research to respond to the sexual and reproductive health and rights of migrants from Venezuela and Central America. Rev Panam Salud Publica. 2023;47:e36. 10.26633/RPSP.2023.36. 36895678 10.26633/RPSP.2023.36 PMC9989552

[7] Sexual and reproductive health of migrants from Venezuela and Central America. J Spec Ser. 2023. https://www.paho.org/journal/en/special-issues/sexual-and-reproductive-health-migrants-venezuela-and-central-america.

[8] WHO Director-General’s opening remarks at the media briefing on COVID-19 - 11 March 2020. https://www.who.int/dg/speeches/detail/who-director-general-s-opening-remarks-at-the-media-briefing-on-covid-19---11-march-2020. Cited 16 Apr 2020. Accessed 16 Apr 2020.

[9] HRP at 50: sexual and reproductive health and rights in epidemic and pandemic preparedness and response. https://www.who.int/publications-detail-redirect/9789240075122. Cited 19 Jul 2023. Accessed 19 July 2023.

[10] Kouanda S, Nahyuha Chomi E, Kim C, Jen S, Bahamondes L, Cecatti JG, et al. Health systems analysis and evaluation of the barriers to availability, utilisation and readiness of sexual and reproductive health services in COVID-19-affected areas: a WHO mixed-methods study protocol. BMJ Open. 2022;12:e057810. 10.1136/bmjopen-2021-057810. 35649598 10.1136/bmjopen-2021-057810 PMC9160592

[11] Cecatti JG, Bahamondes L, Ali M, Alangea DO, Brizuela V, Nahyuha Chomi E, et al. Issues related to pregnancy, pregnancy prevention and abortion in the context of the COVID-19 pandemic: a WHO qualitative study protocol. BMJ Open. 2022;12:e063317. 10.1136/bmjopen-2022-063317. 36202583 10.1136/bmjopen-2022-063317 PMC9539649

[12] R&D Blueprint and COVID-19. https://www.who.int/teams/blueprint/covid-19. Cited 20 Jul 2023. Accessed 20 July 2023.

[13] Mitigating the COVID-19 outbreak through global data sharing. https://www.who.int/teams/health-care-readiness/covid-19/data-platform/mitigating-the-covid-19-outbreak-through-global-data-sharing. Cited 20 Jul 2023. Accessed 20 July 2023.

[14] A Coordinated Global Research Roadmap. https://www.who.int/publications/m/item/a-coordinated-global-research-roadmap. Cited 25 Feb 2026. Accessed 25 Feb 2026.

[15] Unity Studies: Early Investigation Protocols. https://www.who.int/emergencies/diseases/novel-coronavirus-2019/technical-guidance/early-investigations. Cited 20 Sept 2023. Accessed 20 Sept 2023.

[16] Generic protocol: a prospective cohort study investigating maternal, pregnancy and neonatal outcomes for women and neonates infected with SARS-CoV-2, 1 November 2022. https://www.who.int/publications-detail-redirect/WHO-2019-nCoV-pregnancy-and-neonates-2022.1. Cited 19 Jul 2023. Accessed 19 Jul 2023.

[17] Allotey J, Fernandez S, Bonet M, Stallings E, Yap M, Kew T, et al. Clinical manifestations, risk factors, and maternal and perinatal outcomes of coronavirus disease 2019 in pregnancy: living systematic review and meta-analysis. BMJ Br Med J. 2020;370:m3320. 10.1136/bmj.m3320. 32873575 10.1136/bmj.m3320 PMC7459193

[18] Allotey J, Chatterjee S, Kew T, Gaetano A, Stallings E, Fernandez-Garcia S, et al. SARS-CoV-2 positivity in offspring and timing of mother-to-child transmission: living systematic review and meta-analysis. BMJ-Br Med J. 2022;376:11. 10.1136/bmj-2021-067696. 10.1136/bmj-2021-067696 PMC8924705 35296519

[19] Sheikh J, Lawson H, Allotey J, Yap M, Balaji R, Kew T, et al. Global variations in the burden of SARS-CoV-2 infection and its outcomes in pregnant women by geographical region and country’s income status: a meta-analysis. BMJ Glob Health. 2022;7:e010060. 10.1136/bmjgh-2022-010060. 36368768 10.1136/bmjgh-2022-010060 PMC9659713

[20] Giesbers S, Goh E, Kew T, Allotey J, Brizuela V, Kara E, et al. Treatment of COVID-19 in pregnant women: a systematic review and meta-analysis. Eur J Obstet Gynecol Reprod Biol. 2021;267:120–8. 10.1016/j.ejogrb.2021.10.007. 34768118 10.1016/j.ejogrb.2021.10.007 PMC8527829

[21] Schue JL, Singh P, Fesshaye B, Miller ES, Quinn S, Karron RA, et al. Vaccine decision-making among pregnant women: a protocol for a cross-sectional mixed-method study in Brazil, Ghana, Kenya and Pakistan. Gates Open Res. 2024;8:94. 10.12688/gatesopenres.16280.2. (Cited 2 Mar 2026). 39429544 10.12688/gatesopenres.16280.2 PMC11489405

[22] Costa ML, Souza RT, Pacagnella RC, Bento SF, Ribeiro-do-Valle CC, Luz AG, et al. Brazilian network of COVID-19 during pregnancy (REBRACO: a multicentre study protocol). BMJ Open. 2021;11:e051284. 10.1136/bmjopen-2021-051284. 34921076 10.1136/bmjopen-2021-051284 PMC8685531

[23] Costa ML, Pacagnella RC, Guida JP, Souza RT, Charles CM, Lajos GJ, et al. Call to action for a South American network to fight COVID-19 in pregnancy. Int J Gynaecol Obstet. 2020;150:260–1. 10.1002/ijgo.13225. 32412120 10.1002/ijgo.13225 PMC9087527

[24] University of Birmingham. COVID-19 in pregnancy (PregCOV-19LSR). Univ. Birm. https://www.birmingham.ac.uk/research/who-collaborating-centre/pregcov/index.aspx. Cited 20 Jul 2023. Accessed 20 Jul 2023.

[25] Tang K, Gaoshan JJ, Ahonsi B. Sexual and reproductive health (SRH): a key issue in the emergency response to the coronavirus disease (COVID-19) outbreak. Reprod Health. 2020;17:3. 10.1186/s12978-020-0900-9. 32326943 10.1186/s12978-020-0900-9 PMC7179791

[26] Naqvi S, Saleem S, Naqvi F, Billah SM, Nielsen E, Fogleman E, et al. Knowledge, attitudes, and practices of pregnant women regarding COVID‐19 vaccination in pregnancy in 7 low‐ and middle‐income countries: an observational trial from the Global Network for Women and Children’s Health Research. BJOG. 2022;129(12):2002–9. 10.1111/1471-0528.17226. 35596701 10.1111/1471-0528.17226 PMC9347929

[27] Naqvi S, Naqvi F, Saleem S, Thorsten VR, Figueroa L, Mazariegos M, et al. Health care in pregnancy during the COVID-19 pandemic and pregnancy outcomes in six low- and-middle-income countries: evidence from a prospective, observational registry of the Global Network for Women’s and Children’s Health. BJOG Int J Obstet Gynaecol. 2022;129:1298–307. 10.1111/1471-0528.17175. 10.1111/1471-0528.17175 PMC9111322 35377514

[28] Charles CM, Amoah EM, Kourouma KR, Bahamondes LG, Cecatti JG, Osman NB, et al. The SARS-CoV-2 pandemic scenario in Africa: what should be done to address the needs of pregnant women? Int J Gynecol Obstet. 2020;151:468–70. 10.1002/ijgo.13403. 10.1002/ijgo.13403 PMC9087788 33020902

[29] Show KL, Thwin T, Aye NS, Htun ZT, Wai KT. Ethical oversight of implementation research in rural settings of a developing country during the COVID-19 outbreak. Global Bioethics Enquir J. 2021;9:20–7. 10.38020/GBE.9.1.2021.20-27.

[30] Charles CM, Munezero A, Bahamondes LG, Pacagnella RC. Comparison of contraceptive sales before and during the COVID-19 pandemic in Brazil. Eur J Contracept Reprod Health Care. 2022;27:115–20. 10.1080/13625187.2022.2027364. 35156489 10.1080/13625187.2022.2027364

[31] Charles CM, Osman NB, Arijama D, Matingane B, Sitoé T, Kenga D, et al. Clinical and epidemiological aspects of SARS-CoV-2 infection among pregnant and postpartum women in Mozambique: a prospective cohort study. Reprod Health. 2022;19:164. 10.1186/s12978-022-01469-9. 35854384 10.1186/s12978-022-01469-9 PMC9297548

[32] Souza RT, Cecatti JG, Pacagnella RC, Ribeiro-Do-Valle CC, Luz AG, Lajos GJ, et al. The COVID-19 pandemic in Brazilian pregnant and postpartum women: results from the REBRACO prospective cohort study. Sci Rep. 2022;12:11758. 10.1038/s41598-022-15647-z. 35817818 10.1038/s41598-022-15647-z PMC9272878

[33] To NM, Dang HT, Nguyen TVA, Nguyen TTH. Common mental disorders among pregnant women during the Covid-19 pandemic: A scoping review. J Med Res. 2022;161:143–62. 10.52852/tcncyh.v161i12E11.1185.

[34] Ali M, Kiarie J. Pandemic lessons: protecting sexual and reproductive health during global health emergencies. Reprod Health. 2026;22:277. 10.1186/s12978-026-02273-5. 41699709 10.1186/s12978-026-02273-5 PMC12910720

[35] Sawyer A, Aicken C, Huber JW, Vera J, Williams D, Ali M, et al. Contraceptive and sexual health services during the COVID-19 pandemic and recovery: a mixed-methods study in England. Reprod Health. 2026;22:276. 10.1186/s12978-025-02184-x. 41612449 10.1186/s12978-025-02184-x PMC12857002

[36] Ogum D, Maya ET, Modey E, Manu A, Torpey K. Family planning and abortion service availability and utilisation during the COVID-19 pandemic in Ghana. Reprod Health. 2025;22:234. 10.1186/s12978-025-02122-x. 41261426 10.1186/s12978-025-02122-x PMC12632033

[37] Ali M, Kapustianyk G, Seuc AH, Bahamondes L, Cecatti JG, Zotareli V, et al. Evidence on sexual and reproductive health service delivery during and post COVID-19: a multi-country facility assessment. Reprod Health. 2025;22:233. 10.1186/s12978-025-02185-w. 41261430 10.1186/s12978-025-02185-w PMC12632035

[38] Sothornwit J, Lumbiganon P, Jampathong N, Rungreangkulkij S, Kaewjanta N, Kim C, et al. Impact of COVID-19 pandemic on family planning and sexual transmitted infection services in Thailand: results from WHO survey. Reprod Health. 2025;22:165. 10.1186/s12978-025-02092-0. 41029698 10.1186/s12978-025-02092-0 PMC12482536

[39] Zhang H, Wang H, Xie X, Xiao AY, Ali M, Kim C, et al. Evaluating health system barriers to sexual and reproductive health service delivery during the COVID-19 pandemic in China: a mixed-methods study. Reprod Health. 2025;22:162. 10.1186/s12978-025-02093-z. 40983932 10.1186/s12978-025-02093-z PMC12455775

[40] Okwaro F, Mohiddin A, Temmerman M. The psychosocial impact of COVID-19 mitigation measures on frontline staff providing sexual health and family planning services in Kenya: a mixed-methods study. Reprod Health. 2025;22:160. 10.1186/s12978-025-02089-9. 40940648 10.1186/s12978-025-02089-9 PMC12433001

[41] Bahamondes L, Cecatti JG, Munezero A, Soeiro RE, Fernandes KG, Haddad SM, et al. Disruption and recovery of family planning, contraception and other sexual and reproductive health services in Brazil with COVID-19 pandemic: a mixed methods approach. Reprod Health. 2025;22:143. 10.1186/s12978-025-02088-w. 40781681 10.1186/s12978-025-02088-w PMC12333048

[42] Perrotta C, Downey V, Elabbasy D, Ingram C, Lo C, Naseer A, et al. Remote training for strengthening capacity in sexual and reproductive health and rights research: a systematic review. BMC Public Health. 2023;23:1964. 10.1186/s12889-023-16851-w. 37817136 10.1186/s12889-023-16851-w PMC10566165

[43] Palmero A, Carracedo S, Cabrera N, Bianchini A. Governance frameworks for COVID-19 research ethics review and oversight in Latin America: an exploratory study. BMC Med Ethics. 2021;22:147. 10.1186/s12910-021-00715-2. 34742278 10.1186/s12910-021-00715-2 PMC8571668

[44] Doroshow D, Podolsky S, Barr J. Biomedical research in times of emergency: lessons from history. Ann Intern Med. 2020;173:297–9. 10.7326/M20-2076. 32379854 10.7326/M20-2076 PMC7233188

[45] Alirol E, Kuesel AC, Guraiib MM, dela Fuente-Núñez V, Saxena A, Gomes MF. Ethics review of studies during public health emergencies - the experience of the WHO ethics review committee during the Ebola virus disease epidemic. BMC Med Ethics. 2017;18:43. 10.1186/s12910-017-0201-1. 28651650 10.1186/s12910-017-0201-1 PMC5485606

[46] Halstead SB, Tugwell P, Bennett K. The International Clinical Epidemiology Network (INCLEN): a progress report. J Clin Epidemiol. 1991;44:579–89. 10.1016/0895-4356(91)90222-u. 2037863 10.1016/0895-4356(91)90222-u

[47] Gómez Restrepo C, Muñoz NS, Ruiz AJ, Lanas F. Latin American Clinical Epidemiology Network Series - Paper 1: The Latin American Clinical Epidemiology Network “LatinCLEN.” J Clin Epidemiol. 2017;86:71–4. 10.1016/j.jclinepi.2016.10.002. 27789317 10.1016/j.jclinepi.2016.10.002

[48] Council on Health Research for Development - COHRED | Making health. Counc. Health Res. Dev. - COHRED. https://www.cohred.org. Cited 1 Feb 2024. Accessed 1 Feb 2024.

